# The missing voices in the conscientious objection debate: British service users’ experiences of conscientious objection to abortion

**DOI:** 10.1186/s12910-023-00934-9

**Published:** 2023-08-21

**Authors:** Becky Self, Clare Maxwell, Valerie Fleming

**Affiliations:** 1 Exchange Station, Tithebarn Street, Liverpool, L2 2QP UK; 2 School of Health, 81 Tithebarn St, Liverpool, L2 2ER UK

**Keywords:** Conscientious objection, Abortion, Termination, Pregnancy, Found poetry, Healthcare, GP, Abortion access

## Abstract

**Background:**

The fourth section of the 1967 Abortion Act states that individuals (including health care practitioners) do not have to participate in an abortion if they have a conscientious objection. A conscientious objection is a refusal to participate in abortion on the grounds of conscience. This may be informed by religious, moral, philosophical, ethical, or personal beliefs. Currently, there is very little investigation into the impact of conscientious objection on service users in Britain. The perspectives of service users are imperative in understanding the real-world consequences and potential impact of conscientious objection and should be considered when creating and reviewing policies and guidelines. This research provided a platform for women and those who can become pregnant to share their experiences and opinions at a time when these voices are largely excluded in the great tradition of Western political philosophy and law-making processes.

**Method:**

Five service users were interviewed using a narrative interview approach to uncover their abortion journeys and experiences of conscientious objection.

**Findings:**

The findings were presented as found poems and uncovered that doctors are not always: informing service users that they have a conscientious objection to abortion, giving service users enough information to access abortion (indirect referral), treating them non-judgmentally, and providing medically correct information. Service users did not experience burdens such as long waiting times and were still able to access legal abortion. However, service users did experience negative emotional effects, as they were often left feeling scared, angry, and hopeless when they were not referred and/or were mistreated.

**Conclusions:**

Findings indicate that conscientious objection could work in practice. However, it is currently failing some individuals on an emotional level, as not all doctors are adhering to guidelines. Conscientious objection in Britain needs to be addressed, to ensure service users receive fair, impartial, non-judgmental care.

**Supplementary Information:**

The online version contains supplementary material available at 10.1186/s12910-023-00934-9.

## Introduction

Abortion is regarded as a safe and simple medical procedure [1, 2]. It was made available under certain circumstances in Britain with the introduction of the 1967 Abortion Act [3]. It is however illegal when these criteria are not met under the Offences Against the Persons Act in England and Wales [4], and common law in Scotland [5]. With this Act came the introduction of the so-called ‘conscience clause’. This clause allows individuals including health care professionals to object to participating in abortion on grounds of conscience, reflecting the morally contentious nature of abortion. The introduction and potential impact of this clause have been debated within many disciplines and by numerous scholars including lawyers, philosophers, and ethicists [6, 7].

Debates surrounding conscientious objection often present a clash of rights between the healthcare professional and the service user [8]. Thus, radical scholars and thinkers believe the conscience clause should be abolished. Countries that adopt this mentality include Sweden and Finland [9]. In Sweden there is no mention of conscientious objection in the abortion law, whereas in Finland conscientious objection is legally outlawed in its abortion act [9]. However, this ‘zero sum’ presentation of conscientious objection has been challenged [10], and liberal feminist writers present conscientious objection as a balancing act rather than a clash of rights [11]. It has been argued that health care professionals can object without becoming a barrier for service users to access abortion [10, 12]. One way this can be achieved is by introducing what Brock termed the ‘conventional compromise’. In this approach, health care professionals are required to inform the service user about abortion and refer them to another health care professional who is willing to provide the service. They are able to object in this way if the referral does not impose an unreasonable burden on the service user [13].

Furthermore, British guidelines stipulate that when doctors object they must inform the service user of their objection without passing judgment. They must inform the service user that they are able to see another health care professional and they have to provide enough information for the service user to make another appointment [14]. These requirements are not reflected in the Abortion Act (1967). Doctors must not obstruct service users from accessing abortion or leave them with nowhere to turn [14]. In Britain doctors self-regulate their objections, this makes it hard to establish how conscientious objection is playing out in practice, and whether it is impacting British service users and other health care professionals.

There is little information on how conscientious objection is playing out in practice and the impact it is having on service users. British research from the service users’ perspectives indicates that objections that go against guidelines are occurring, as general practitioners (GPs) are not always informing service users of their objections [15, 16] and they do not always inform service users that they are able to discuss their treatment with another doctor [15–17]. Moreover, service users are not always provided with sufficient information on how to obtain an abortion [15, 16], and in some instances are provided with incorrect or morally loaded information. For example, a 17-week pregnant service user in Scotland was told by a GP that “she was “too late” for a termination, as the foetus was a “baby now”” (17, p. 105). Conscientious objection that goes against guidelines has been described by service users as “traumatic” (15, p.17), and has the potential to alienated service users [16]. Thus, existing literature shows that practice that does not adhere to guidelines is having a negative emotional impact on British service users and is causing delays in accessing services [15, 16]. However, it is important to note that the findings of others [15–17] are outdated, focus on small geographical areas, and do not set out to uncover conscientious objection, as they focus on assessing service delivery. Thus, their contributions to the conscientious objection debate are limited.

## Method

### Theoretical Framework

Current research on conscientious objection approaches the issue by researching healthcare professionals’ views and experiences and discussing the ethical, legal, philosophical, and financial aspects of conscientious objection. However, the impact conscientious objection has on service users is of utmost importance [18, 19] and has not been researched adequately from first-hand accounts of service users. A liberal feminist theoretical framework was employed to combat this issue and to challenge the unequal power dynamic faced by women and pregnant individuals within society. This research provided a platform for service users to share their experiences and thoughts, at a time when their voices are largely excluded in the great tradition of Western political philosophy and law-making processes [20–23]. Thus, service users had the opportunity to have their say on matters that have the potential to impact their reproductive rights, whilst challenging the systematic devaluation of their voices in the context of patriarchy [24], and within the paternalistic power dynamics of healthcare. Attempts have been made to include the voices and experiences of women in some areas of law and policy creation [25], this research takes a liberal feminist perspective to extend this practice to conscientious objection to abortion.

The decision to implement a liberal feminist theoretical framework meant power and law were at the heart of the research, as liberal feminists posit that change and equality can be achieved by legal reform [26]. Thus, the impact that conscientious objection law and guidelines have on service users was seen as paramount. Also, liberal feminist theory utilised working within the current legal infrastructure rather than overhauling the existing mechanisms and power dynamics in place within the legal system [26]. This meant policy implications drawn from the research offer practical solutions to the potential issues faced by service users in Britain.

### Research questions

The research aimed to answer the following question:

Has the fourth clause of the 1967 Abortion Act (conscience clause) affected service users’ reproductive rights concerning access and experience of abortion? and if yes then how?

### Participants

This article presents the experiences of five service users who experienced conscientious objection to abortion. They were recruited using purposive sampling from March 2020 until April 2021. Service users self-selected to advertising material on the platform ‘Call For Participants’ [27], paid Facebook advertising, and online forums. The inclusion criteria were anyone over the age of 18 who had accessed an abortion in the United Kingdom (UK). Individuals could not take part if they abused alcohol/drugs, had a serious mental health condition, or if they would become extremely distressed when talking about abortion. The criteria were later revised to focus the recruitment. Individuals over 18 who fit the following categories could participate: 1. Attempted to access abortion in the UK but were refused for a non-medical reason. 2. Experienced a health care professional refusing to participate in their abortion, for a non-medical reason. 3. Experienced an abortion referral to another healthcare professional for a abortion for a non-medical reason. Table 1 shows the demographic information of the five service users presented in this article. Pseudonyms were allocated to each service user. All service users identified as cis-female.

**Table 1:** Participant Demographics and Abortion Gestation and Type

Participant	Age at interview	Age when aborting	Location	Ethnicity	Occupation	Gestation in weeks	Type of abortion
Maria	26	24	Urban South England	Italian	Teaching Assistant	14	Surgical or Vacuum
Katie	23	22	Urban Wales	Black	Unemployed	8	Medical
Jess	51	25	Urban South England	White British	Unemployed	-	Surgical
Emma	21	18	Rural South England	British	Student	1st trimester	Medical
Charlie	38	38	Urban South England	White British	Art Therapy Support Worker	8	Medical

These five participants took part in the first author’s Ph.D. research project which consisted of 25 service users overall [6]. The intention of the Ph.D was to uncover service users abortion journeys (those who had and had not experienced conscientious objection), uncover the potential impact of conscientious objection on service users, and understand service users views on conscientious objection. These five service users were selected for this article as they were the only service users who had experienced a clear case of conscientious objection.

### Study Design

#### Data Collection

A hybrid interview approach was utilised, this combined both narrative and semi-structured interview approaches. The data presented in this article was derived from the narrative element of the interviews. Table 2 depicts the interview guide for the narrative element of the interviews.

**Table 2:** Interview Guide

Please tell me about your abortion journey in as little or as much detail as you would like. Feel free to stop at any time.
**General prompts for those who do not benefit from a narrative approach**: Age? Number of abortions? Location? What stage of the pregnancy? What type of abortion? Did you feel supported by staff?
**Prompts regarding conscientious objection for those who do not benefit a narrative approach**: How did this impact you? Time? Emotion? Financial element? Did this change your relationship with the healthcare professional? How? When the healthcare professional objected what happened next? Quick process? Referral process?

A pilot study was carried out with friends and colleagues who had accessed an abortion, to ascertain the appropriateness and understandability of the interview guide. The first author carried out all the interviews herself in English. Service users’ socio-demographic data were collected prior to the interview via the research website [28].

The interviews were conducted over Zoom, MS Teams, and the telephone. Service users were given the choice of interview mode. The decision to use these modes was made in light of the Covid-19 pandemic, and by deliberating which mode to use by creating a framework [29]. Interviews, therefore, took place in the researcher’s and service users’ own environments.

The interviews were recorded on an encrypted recording device and transcribed verbatim. All of the service users provided written informed consent via the consent form on the research website [28]. Informed consent was re-confirmed verbally at the beginning of each interview. Ethical approval was obtained from the Liverpool John Moores University (UK) ethics review committee (20/NAH/001). Interviews lasted between 35 and 51 min (this included the discussion around their views on conscientious objection). Service users received £30 (love to shop gift vouchers) through the post for sharing their time and experiences.

### Data Analysis

Found poems were constructed for each participant by the first author over a period of three months. The transcripts were read numerous times, and nuggets [30], key phrases, and sentences, that developed and demonstrated individuals’ experiences [31], and could be seen as core to service users’ journeys, were highlighted. Many of these nuggets were moving, powerful, meaningful, and thought-provoking [32]. The nuggets were then transferred onto another sheet of paper, and arranged to form a poem. This was not done linearly, the audio recordings and transcripts were referred back to and the poems were revised throughout, to gain a sense of meaning, rhythm, and understanding [30]. This process is described as “intuitively sorting out words, phrases, sentences, passages that synthesize meaning from the prose” (33, p. 547). This task of “removing material”, has been deemed a process of analysis that enables researchers to better understand and express participant responses (34, p.6). The co-authors reviewed the poems to ensure they represented the data and service users’ experiences. This method of analysis provided a platform for service users to share their experiences of conscientious objection thus reflecting the liberal feminist underpinnings of the research. Liberal feminism also informed the research discussion as unequal gender power dynamics, power within the health service, and guidelines were presented as central to the service users’ experiences.

### Reflexivity

The research teams’ preconceptions, identities, and education inevitably impacted all stages of the research process. A reflexive diary was kept throughout to note opinions, emotions, and research decisions. The research team had different identities, experiences, and perspectives concerning abortion and conscientious objection. This meant that assumptions and perspectives were challenged throughout. The research team consisted of three white women in academia, two had practiced midwifery and one had a background in sociology with no clinical experience. One was non-religious and pro-choice, one was a non-practicing Roman Catholic and pro-choice, and one a practicing Roman Catholic and anti-abortion.

The first authors’ opinions on conscientious objection developed throughout the research process. Before reading any literature on conscientious objection she took a zero-sum approach [10] believing that conscientious objection is incompatible with service users’ rights. After reading copious amounts of literature and carrying out the interviews she concluded that conscientious objection is not necessarily anti-feminist and should be allowed to protect healthcare professionals’ human rights, so long as service users’ rights remain paramount. However, she treats this opinion with great caution as she believes conscientious objection is fraught with issues and regulation is necessary. The co-authors’ views on conscientious objection and how they were shaped by and in turn shaped another study have previously been published [35].

## Findings

The following found poems present the abortion journeys’ of five service users who experienced conscientious objection in Britain.

Charlie.

I was very young,

twenty. I was attacked.

I did all the wrong

things. I bathed,

got rid of evidence. I panicked

because I knew my attacker.

It was a big scramble

to get everything done.

The process

itself was traumatic.

Not because of the professionals

involved, because of the circumstances.

The first GP

I went to. She did tell me that

she didn’t agree

with it, because of her background,

her beliefs.

It was handled

in a very sensitive way. I didn’t

feel judged.

I respected her decision

and she respected mine. The right

amount of balance.

She said I’d need to speak

to another GP.

I wouldn’t have to wait long

for the second referral, to go to a different doctor.

Within a week it was resolved.

Then I was referred

to The Brook service

I had the medical

procedure. I was eight weeks

so I just took the tablets.

I had support from Hayden,

and support from The Brook centre.

That was my journey.

Emma.

I was eighteen. I had two

different tests. Saying two different

things.

We have a family doctor, she knows my mum.

I went there to confirm.

I was really scared

she was gonna say something.

It was positive.

Right there and then

I just said

I didn’t want to keep it.

She said my mum

needs to know.

She needs

to have her say.

That it wasn’t the only option.

I knew what I needed

to do, I didn’t want anyone

else influencing my decision.

I asked “where

do I go from now?” “Can you

refer me?” “Give me a different

Dr?” She said

“No everyone’s busy

just look online”

she didn’t really even give me any information

or websites.

It was very

discouraging. I was thinking

am I making the right decision?

She went about it

the wrong way.

I’m not sure if hers wasn’t relevant for religious views.

I was really clueless. Do I

go to the GP? Do I call?

Do I have

to pay?

She didn’t give me

any further information.

I had to call

and specifically

ask for someone else. I made an appointment

two weeks later.

She was helpful. Let me know

my options.

What would happen

if I do. What would happen if I don’t.

She was able to refer me.

I had the pills.

It was quite

early on. Easier just to

pop a pill and be done.

Jess.

I was around 25. I was experiencing

homelessness and other issues.

I had a long term

boyfriend at the time. I found out

I was pregnant. He wasn’t

happy. It was more along the lines of threats.

I could have been killed.

He wasn’t joking.

I decided I had

to do it. If I can’t

look after myself, I can’t

look after a child.

I went to make an appointment

with the doctor. The appointment came

around, I didn’t sleep

the night before.

I said “I’m pregnant

and I’d like to have an abortion”.

“I can’t do that for you.”

That was it.

I’m just assuming religious reasons.

But I could be wrong.

I started crying.

I was already feeling

really distressed. I just wanted

to get out of there.

It was absolutely horrific.

Thinking about it just makes

me angry.

You have the means

to help me. And you don’t

want to.

After that it was this awkward

silence. He was giving

me this half-assed sympathetic look.

He just didn’t do anything.

I just said “ok

thank you”, and walked

out, up to the reception in tears.

She had to rebook

I had to wait again. I walked out

in absolute disbelief.

Eventually everything

did go ahead. It was surgical.

They were nice. They were

friendly. They weren’t judgemental.

I think it was a Marie Curie clinic.

It’s not an easy

choice to make. If it had been different

circumstances, I wouldn’t

have chosen an abortion.

I just felt like my hands

were tied.

Katie.

I wanted to do

the abortion. I was not comfortable

to keep the kid.

It’s not like I was stable

financially. I was also doing studies.

He was the main

Dr in the hospital.

He refused

to participate. It was his personal

beliefs. He felt it was like killing.

He said he’s a religious man

his faith

could not allow him.

I tried convincing him, giving

him more money.

He refused completely.

I felt bad.

He tried convincing me

of not doing it. Maybe

I might not be able to have a kid

later on in life.

Maybe there was an effect

on the womb. Maybe I’d be feeling

like I killed someone.

I had second thoughts

at some point. The need

of wanting

to get rid

of the kid was much more than this feeling.

I had no idea

where to consult.

I didn’t want to have the later consequences

of having the abortion in the wrong way,

the wrong place.

I wanted him

to help me

even if he couldn’t himself.

He referred me. He had no

other choice. I went to BPAS

after two days.

After consulting

I did the abortion there.

I was two months. In the end

I was just given some pills.

Maria.

My experience

was in the summer of 2018.

It was from a pregnancy

with a long term partner.

I found out in Italy

my home country. I decided to go back

to England

because of religious conflict

conscientious objection is a lot more common there.

So I returned

to the UK. Immediately I researched

the options.

I pretty quickly decided

I wanted to end the pregnancy. I was around

fourteen weeks.

I didn’t have a very positive

experience with my GP. They weren’t

on board. My answers were

very much contested.

Devil’s advocate.

I was aware they were strongly religious.

I was not referred.

I was scared

that’s going to be the reaction everywhere.

I was upset

having to answer all those very personal

questions. It was quite

frustrating.

I then contacted another reproductive clinic.

I found them to be a lot more supportive,

less judgemental.

I ended up going through with them.

It was surgical.

## Discussion

By utilising found poetry service users’ abortion journeys and experiences of conscientious objection were humanised and presented holistically. The following issues are discussed in the sections below: accessing abortion services, transparency from doctors, how doctors navigate conscientious objection, and the impact of conscientious objection on service users. Unequal gender power dynamics, power within healthcare, a liberal legal stance, and policy recommendations informed these discussions, due to the liberal feminist standpoint of the research.

### Accessing abortion services

It can be argued that the conscientious objection clause is often bypassed in Britain as 77% of abortions in England and Wales in 2021 were performed by NHS-funded private clinics, such as the British Pregnancy Advisory Service and MSI Reproductive Choices [36]. This could indicate that service users are self-referring when accessing abortion and are not approaching GPs. If service users are self-referring it is unlikely that they will come into contact with an objector, as it would be unusual for a health care professional working at an NHS-funded private clinic to conscientiously object. However, this statistic does not take into account those service users who have been referred to these services by their GP, or those who have sought out these services themselves after a lack of referral from their GP or another healthcare facility.

Furthermore, in the current study one’s GP was viewed as an appropriate access route to abortion, as the majority of service users (Maria, Jess, Emma, Charlie) accessed their abortion in this way. Service users who visited their GP initially saw this as the most logical route, commenting without question and as a matter of fact, that the first health care professional they approached was their GP.

However, service users also attempted to access abortion via non-conventional access routes, due to a lack of awareness. Katie attempted to access abortion through a public hospital, she believed she had to pay for an abortion, and she commented that she did not know how to access safe abortion without a referral. None of the service users in the current study were aware that they could self-refer to access an abortion, indicating there is an unequal power dynamic between doctors and service users; as service users relied on doctors to access vital care, and struggled without their guidance. This is despite claims within the literature that there is a plethora of advice for service users online around abortion, meaning access should be simple and safe [37]. Thus, the conscience clause may not be bypassed to the extent the statistics – on the numbers of abortions procured via NHS-funded private clinics [36] – infer (as previously outlined). More research in this area is needed to confirm this claim. If confirmed more public education on self-referral routes should be introduced.

### Transparency

Whether doctors should inform service users of their objections, go into detail, and explain their objections, has been debated in the literature [13, 38, 39]. Guidelines stipulate that in Britain doctors must inform the service user that they do not provide abortion care without causing distress. They can discuss the reason for their objection if they wish, so long as they do not cast judgement [14].

Charlie commented that her GP informed her that she had an objection, as she stated “she did tell me that she didn’t agree with it, because of her background, her beliefs”. Thus, Charlie was aware that her GP was objecting on non-medical grounds because of her beliefs. The GP’s actions were in line with policy guidelines [14]. Charlie commented that she respected her GPs decision, this clear communication and reasoning could have aided this. Katie was also informed by the doctor why he could not be involved in her abortion “[i]t was his personal beliefs. He felt it was like killing. He said he’s a religious man. His faith could not allow him.” This was followed by an attempt to change Katie’s trajectory to prevent her from having an abortion. Thus, he went further than outlining and explaining his own beliefs and went against guidelines [14].

However, not all service users understood why their doctor objected, though they knew their objection was not based on medical reasons or concerns. For example, Emma stated: “I’m not sure if hers [GP] wasn’t relevant for religious view.“ This indicated that Emma couldn’t pinpoint why her GP was objecting, though she believed it was not due to religious reasons. This opinion may have been fuelled by the fact that Emma’s GP was pushing for her mother to be involved in the decision-making process. Emma may have believed the GPs objection was based on her (Emma’s) age, and the fact that the GP knew her and her family, rather than religion (all of these reasons can be the basis of a conscientious objection.) Moreover, Jess also assumed why her health care professional was objecting, as she stated “I’m just assuming religious reasons. But I could be wrong.” Jess reached this conclusion as her GP sat in silence after he commented that he could not be involved in her abortion. This reinforced the assumed association between religion and conscientious objection that is present in the media and some areas of academia (40–41). Lastly, Maria commented that she was aware that her GP was strongly religious, although she did not elaborate on how she knew this.

Doctors need to be more transparent in their decision-making, by stating they have an objection in a non-judgmental way, to prevent confusion from the service users’ perspectives. Many service users in the current study were left perplexed when the doctor did not explain why they were making certain decisions, this could lead to consequences for service users. Moreover, lack of transparency, oversharing one’s opinion, and passing judgement went hand in hand with improper treatment that went against guidelines. More education and regulation are needed to ensure doctors adhere to these guidelines. Conscientious objection could be regulated using a harm reduction based approach where objecting doctors detail in advance how they will ensure the service user can access an abortion when they wish to object [42]. However, such an approach is problematic as doctors have to pre-empt their objection and must self-regulate their actions when objecting. More research is needed in this area to develop a less problematic way of regulating conscientious objection.

### Doctors navigating conscientious objection

In Britain objecting doctors are not only obliged to inform service users of their objection, but to inform them that they can discuss their condition and treatment options with another healthcare professional who can advise them on abortion (in-direct referral). The objecting doctor has to ensure that the service user has enough information to make another appointment to see a non-objecting doctor. If it is not practical for the service user to make arrangements to see another doctor, the objecting doctor must make immediate arrangements for a qualified colleague to refer, treat or advise the service user. Also, doctors must not obstruct service users’ access to abortion [14]. Charlie received care from her GP that went beyond these requirements as Charlie’s GP told her that she could not facilitate her abortion as it was against her beliefs. The GP then went on to refer Charlie to another GP who could provide her with an abortion. Charlie explained that “It was handled in a very sensitive way”.

However, Katie, Jess, Maria, and Emma were not informed that they could speak to another healthcare professional about accessing abortion. Maria inferred that abortion was not outlined as an option by her GP as “[t]hey weren’t on board. My answers were very much contested.“ Similarly, Emma’s GP focused on what her mother would think and that an abortion was not Emma’s only option. When Emma asked her GP to refer her she was informed that “everyone’s busy” (GPs at the clinic) and to “just look online”. Moreover, Jess’s GP would not have a discussion with her, as Jess explained that her GP said “I can’t do that for you.“ [and] “That was it.” Lastly, Katie’s doctor gave her medical misinformation and views from his moral stance, though he did refer her to the British Pregnancy Advisory Service. Katie inferred that it was her assertiveness that pressured the doctor into referring her. Thus, discussing abortion with another healthcare professional was not presented as a legitimate option for Maria, Emma, Jess, or Katie, and the doctors’ actions can be seen as obstructing abortion access. This reflects other findings from England [15, 16], as some objecting GPs avoided discussing the available options with service users, and left service users uninformed.

In sum, service users’ commentary makes it evident that doctors either weren’t aware of their obligation to inform service users that they can see another healthcare professional to discuss obtaining an abortion and not to obstruct access, or doctors were aware and choose not to follow guidelines. Moreover, findings indicated that there needs to be more education for doctors about how they should navigate conscientious objection, and more regulation to ensure they are doing so. As previously noted there is no clear route for successfully regulating conscientious objection.

### Service users navigating conscientious objection

In line with guidelines doctors should not leave service users without anywhere to turn [14]. Charlie’s GP referred her to another GP to provide her with an abortion, and the doctor Katie approached at the hospital referred her to the British Pregnancy Advisory Service. However, Jess, Maria, and Emma had to navigate accessing abortion alone. Emma decided to telephone and book an appointment with a different GP at the same surgery. Jess made an appointment with the receptionist at the same surgery with a different GP, whilst still present at the surgery. This reinforced the perceived importance of GPs that is previously discussed, and the lack of options and education the service users may have had. Maria did not return to her GP, instead, she contacted a reproductive clinic herself, she did not explain why this was not her initial choice. Thus, all service users were able to access an abortion and in this sense, in reality, were not left without anywhere to turn. However, service users often felt isolated and like they had nowhere to turn as discussed in the following section.

In addition, the current stipulate that “if it is not practical for a patient to arrange to see another doctor, you must make sure that arrangements are made for another suitably qualified colleague to take over your role” ([43], para.52). These guidelines are problematic as there is no guidance for doctors to assess if it is practical for the service user to make another appointment. Thus, it is at the doctors’ discretion which furthers the unequal power dynamic between service user and doctor. Although findings showed that service users were able to make subsequent appointments, the lack of referral had consequences on an emotional level (detailed in the section below), which is not adequately considered in the current General Medical Council Code and is exacerbated by the lack of education around accessing abortion. Secondly, the service user may make another appointment and be met with another objecting doctor. This could leave service users believing they have nowhere to turn. Although this did not occur in the study.

### Impact of conscientious objection on service user

How the doctors conducted themselves had a negative emotional impact on four of the five service users who experienced conscientious objection. Charlie was not emotionally impacted by the actions of the GP as she commented “[t]he process itself was traumatic. Not because of the professionals involved, because of the circumstances.” Charlie found the process traumatic and she was raped, meaning she had to navigate rape counselling services while accessing abortion, in addition to the emotional impact of experiencing rape. Charlie was referred and informed effectively and this did not have an emotional impact on her, or alter the type of abortion accessed, as she stated “within a week it was resolved […] I was eight weeks so I just took the tablets.” Thus, indicating successful implementation of the conventional compromise approach [12, 13], as the GP referred and informed the service user.

However, the impact that the doctors’ actions had on Katie, Jess, Maria, and Emma was detrimental to their emotional well-being and added pressure on them to navigate accessing abortion alone. Jess explained that “I started crying. I was already feeling distressed. I just wanted to get out of there. It was absolutely horrific.” It was obvious that the GP’s actions of stating he could not do anything for her and sitting in silence, had a significant negative impact on her emotional well-being, as she described the appointment as “horrific” and it brought her to tears and caused distress.

In addition, Maria was frustrated and concerned with how she would access an abortion as she commented that “I was scared that’s going to be the reaction everywhere. I was upset having to answer all those very personal questions. It was quite frustrating.” Moreover, Emma’s commentary inferred that she was agitated and worried about how to access an abortion as she asked for this information to no avail. She found the lack of information discouraging and questioned whether she should even have an abortion. Thus, indicating the potential impact GPs could have on service users’ decisions. She was still able to access abortion services. Similarly, Katie also questioned whether she should have an abortion “I had second thoughts at some point” and was worried about where she could obtain one “I did not want to have the later consequences of having the abortion in the wrong way, the wrong place. I wanted him to help me even if he couldn’t himself.” Thus, the emotional impact that the doctors’ actions had on these service users was unacceptable and went against current guidelines [43]. These findings reflect those of Finnie, Foy, and Mather [15] as participants in Durham, England commented that objecting GPs caused emotional distress, and Biggs, Kaller, and Ralph [44] showed that there is a positive correlation between barriers to abortion and anxiety symptoms.

However, all of the service users managed to access an abortion promptly. Charlie was referred to another GP within a week, and Katie was referred to the British Pregnancy Advisory Service after two days. Emma had to wait two weeks for another GP appointment, fortunately, this did not impact the type of abortion she accessed, as she was still in her first trimester. Jess and Maria did not comment on how long it took for them to see another healthcare professional. Thus, conscientious objection did not prevent service users from accessing abortion, or seemingly alter how they chose to abort. However, it could have caused unnecessary delays and did have an emotional impact on the majority of service users. Again, this indicates that there needs to be more education and regulation for doctors around conscientious objection, once an appropriate way of regulating conscientious objection in Britain is discovered.

### Limitations

The majority of service users accessed abortion in England (4) and resided in an urban area (4). Those attempting to access abortion in a rural or remote area (1) were underrepresented, as were service users who were based in Wales (1). Similarly, there were no service users who resided in Scotland or Northern Ireland (the original inclusion criteria included Northern Ireland and investigating the impact of The Abortion (Northern Ireland) Regulations 2020) [45], and all the service users were cis-women, despite the attempt to recruit specifically from Northern Ireland and trans/non-binary populations. Also, non-English speaking service users were not recruited due to research budget restrictions. Thus, findings were unable to present a picture of these service users’ experiences and opinions. These findings would have been useful in light of the recent legalisation of abortion in Northern Ireland and the prediction of high numbers of conscientious objectors [46], and the assessment that conscientious objection disproportionately impacts marginalised individuals, those living in socio-economic deprivation, and those residing in rural areas [47]. Although service users were from a variety of ethnic backgrounds, thus findings were not limited to white middle-class women.

Furthermore, it is possible that there was a bias towards negative experiences of conscientious objection or accessing abortion, as service users may have been more likely to come forward and talk about their experiences to offload - as Jess commented – and tell their story. Although, introducing the financial incentive may have reduced this somewhat, as this would have given potential participants another reason to take part. Moreover, the recruitment process may have led to a certain “type” of participant taking part, as recruitment material was posted on online platforms, Facebook paid advertising, and the recruitment website callforparticipants.com [27]. Although, recruiting in this way meant individuals who had continued their pregnancy, or obtained an abortion from a non-traditional route could take part in the research. These service users may not have been reached by recruiting through abortion services such as the British Pregnancy Advisory Service.

## Conclusions

In conclusion, GPs play an important role in service users accessing abortion, as service users had little knowledge of the self-referral routes to abortion services. This creates unnecessary pressure on GPs and indicates that there needs to be more research in this area, and potentially more public education on accessing abortion through self-referral routes. Moreover, findings indicate that conscientious objection could work in practice, as Charlie’s abortion journey exemplified. However, not all doctors are adhering to guidelines, as in some instances service users were not informed of the doctor’s objection, or that they were able to see another healthcare professional. In addition, doctors passed judgement, misinformed service users, and discussed their own moral opinions. Although service users were able to access abortion in a timely manner, they were left feeling scared, angry, and hopeless. Thus, more education and regulation are needed to ensure doctors adhere to guidelines, and more clarity is needed around these. Conscientious objection in Britain needs to be addressed, to ensure service users receive fair, impartial, non-judgmental care.

### Electronic supplementary material

Below is the link to the electronic supplementary material.

Additional File 1: Interview guide

## Data Availability

Data are available from the corresponding author upon reasonable request.

## Abbreviations

BPAS: British Pregnancy Advisory Service
GP: General Practitioner
NHS: National Health Service
UK: United Kingdom
