# Supplementary material for: The missing voices in the conscientious objection debate: British service users’ experiences of conscientious objection to abortion
Source: BMC Med Ethics. 2023 Aug 21;24:65. doi: 10.1186/s12910-023-00934-9 (PMC10441708; doi:10.1186/s12910-023-00934-9)
Supplement: Supplementary file 1 — Additional File 1: Interview guide [file 12910_2023_934_MOESM1_ESM.docx]

Interview guide.

Narrative interview, to determine service users abortion journeys and the impact and experience of conscientious objection.

| **Please tell me about your abortion journey in as little or as much detail as you would like. Feel free to stop at any time.** |
| --- |
| **Prompts for those who do not benefit from a narrative approach:**  Age? Number of abortions? Location? What stage of the pregnancy? What type of abortion? Did you feel supported by staff? |
| **Prompts regarding conscientious objection for those who do not benefit a narrative approach:**  How did this impact you? Time? Emotion? Financial element? Did this change your relationship with the healthcare professional? How? When the healthcare professional objected what happened next? Quick process? Referral process? |
